# Dataset on the geographical distribution of species of the genus *Solanum,* subgenus *Leptostemonum* in Uganda

**DOI:** 10.1016/j.dib.2024.110159

**Published:** 2024-02-13

**Authors:** Carol Mere Kauma, Godwin Anywar, Derick Serunjogi, Esther Katuura, Mary Namaganda

**Affiliations:** a Department of Plant Sciences, Microbiology and Biotechnology, School of Biosciences, College of Natural Sciences, Makerere University, P.O. Box 7062, Kampala, Uganda; b Department of Biology, Faculty of Science and Education, Busitema University, Uganda

**Keywords:** Geo-coordinates, Environment conservation, Diversity

## Abstract

The dataset includes the diversity, occurrence points and a distribution map of species of the subgenus *Leptostemonum* in Uganda. The data was obtained following field surveys carried out in various parts of Uganda. These were guided by distribution data retrieved from Makerere University Herbarium and Flora of Tropical East Africa (FTEA). At each sampling site, species name, altitude and habitat type were recorded. Occurrence points were geocoded using a hand-held GPS (a ≤ 5M Germin S90 GPS). The distribution map was generated using ArcMap 10.7.1 software. The dataset consists of 172 occurrence points representing 18 species of subgenus *Leptostemonum* that occur in Uganda. The data can be used to assess the effect of climate change on the diversity and distribution of these species. The data set is also important for informing resource users, conservationists and policy makers about the biodiversity hotspots of these economically important species.

Specifications Table

**Table:** 

Subject	Biodiversity
Specific subject area	Geography
Data format	Raw, Analyzed
Type of data	Table, Figure
Data collection	Occurrence data were collected during field surveys carried out between July and December 2019 in the different parts of Uganda. At each sampling site, geo-coordinates and altitude were obtained using a handheld GPS (≤ 5M Germin S90 GPS). Habitat type as well as species were also recorded. Using ArcMap 10.7.1 software, the distribution map was generated.
Data source location	Collected: Uganda (all the four floral regions) Stored: Makerere University Herbarium City: Kampala Country: Uganda
Data accessibility	Repository name: Mendeley data Data identification number: 10.17632/r8dbdy7zmt.1 Direct URL to data: https://data.mendeley.com/datasets/r8dbdy7zmt/2

## 1 Value of the Data

• The geo-coordinates and occurrence maps of *Solanum* species have been contributed as new information since the last study carried out in Uganda about 30 years ago [1].
• This nation-wide dataset has given an update of the important sites for the conservation of the rare and endangered species of the genus and also defined future collection points.
• The data brings significant contributions specifically to the immediate resource users and policy makers including; conservationists, plant breeders and ecologists.
• The data may be used to design better management strategies to improve sustainable use in the face of climate change.

## 2 Background

*Solanum* includes three species of economic importance: tomato (*Solanum lycopersicom* L.), potato (*Solanum tuberosum* L.) and eggplant (*Solanum melongena* L.) with species of minor importance such as pea eggplant (*S. torvum* Sw.) and tree tomato (*S. betaceum* Cav.) [2]. In Uganda, the species are of considerable economic and social importance. For instance, 39% of the vegetables consumed locally are from *Solanum* genus [3] but, they have been neglected in research. This study aimed at giving detailed occurrence data, highlighting the diversity and distribution of these species within the different geographical regions of the country. The data is open and reusable for better planning and conservation.

## 3 Data Description

The data set entitled “Diversity and distribution dataset for the species of the Leptostemonum clade in Uganda (version 2)” was uploaded to Mendeley data repository [4] (https://data.mendeley.com/datasets/r8dbdy7zmt/2). In the repository, the uploaded file was named “distribution data” with its content as given below.

**Fig. 1:**
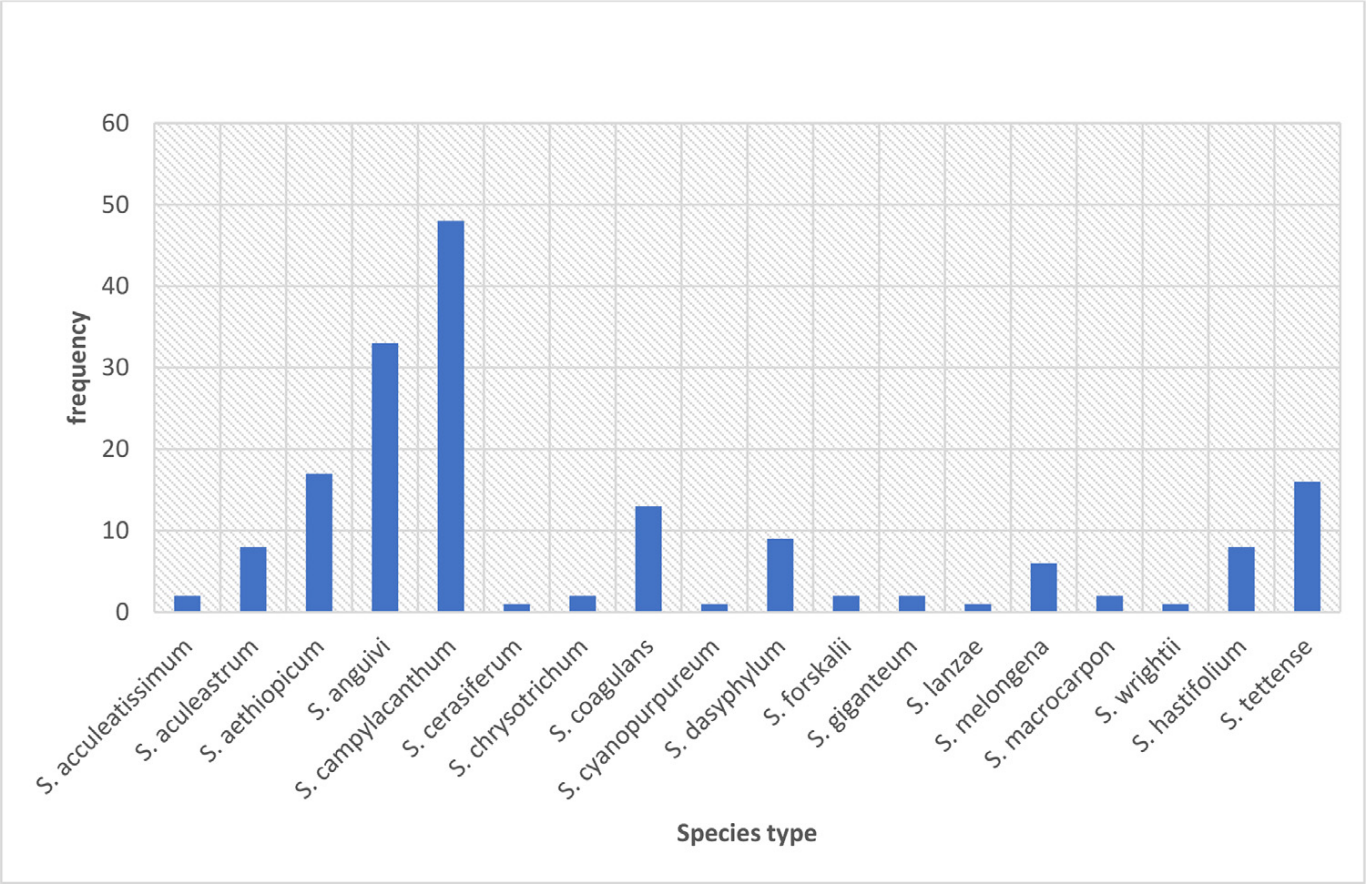
The frequency of each species sampled and georeferenced during the field surveys.

**Fig. 2:**
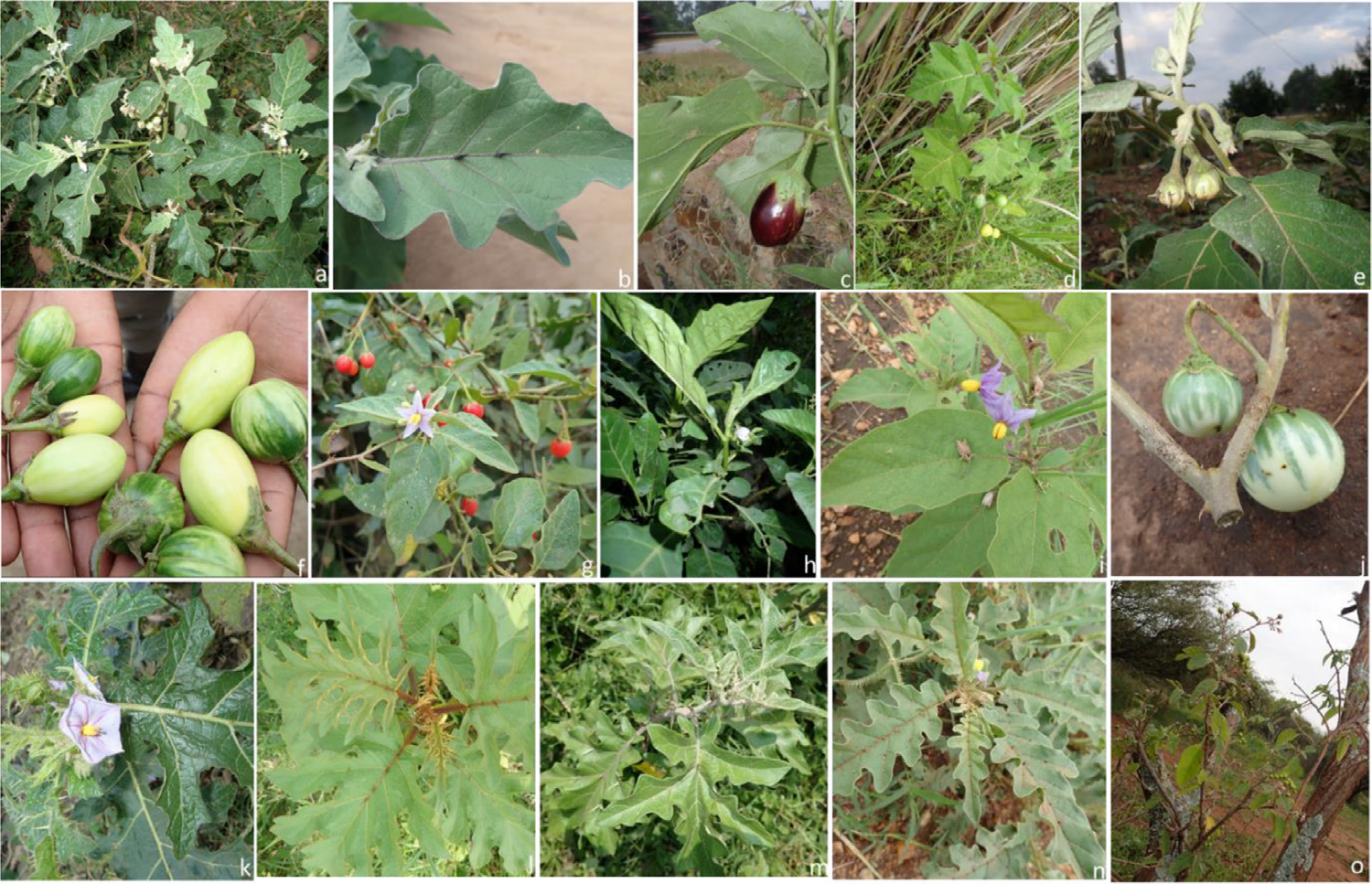
(a – o): Images of *Solanum* species: a. *Solanum anguivi*, b. Leaves of *S. melongena*, c. Fruit of *S. melongena,* d. *S. acculeatissimum*, e. *S. aethiopicum*, f. Fruits of *S. aethiopicum* (Gilo group), g. *S. cyaneopupureum*, h. *S. aethiopicum* (Schum group), i. *S. campylacanthum*, j. Fruits of *S. campylacanthum*, k. *S. dasyphylum,* l. *S. chrysotrichum*, m. *S. aculeastrum*, n. *S. coagulans* and o. *S. tettense*. (Photographs by Carol Mere Kauma).

The data were obtained during our fieldwork surveys carried out between July and December 2019 within the different geographical regions. The data presented includes species occurrence points and the distribution map of the 18 species of subgenus *Leptostemonum* in Uganda. The number of each species sampled and georeferenced have been presented in Fig. 1 and the species distribution per each region presented in Table 1. The species, habitat types as well as the elevation from which they were collected are listed in Table 2. Southwestern Uganda had the highest species numbers collected while Karamoja region had the highest species diversity, with species found in the region restricted within its range. *Solanum campylacanthum* was the most collected species, occurring in all the geographical regions of Uganda. Phenotypic images of some of the 18 species have been presented in Fig. 2 (a – o).

**Table 1:** The species distribution per region in Uganda.

Species	Northern				Western		Eastern			Central	
	West Nile	Acholi	Lango	Karamoja	western	South western	Teso	Elgon	East central	Central 1	Central 2
*S. acculeatissimum*						x					
*S. aculeastrum*						x		x			
*S. aethiopicum*					x	x					
*S. anguivi*					x	x					
*S. campylacanthum*	x	x	x	x	x	x	x	x	x	x	x
*S. cerasiferum*			x								
*S. chrysotrichum*						x					
*S. coagulans*				x							
*S. cyaneopupureum*						x					
*S. dasyphylum*						x					
*S. forskalii*				x							
*S. giganteum*								x			
*S. hastifolium*				x							
*S. lanzea*				x							
*S. macrocarpon*										x	
*S. melongena*						x			x		
*S. tettense*				x							
*S. wrightii*					x						

x denotes species presence

**Table 2:** Species of the subgenus *Leptostemonum* that occur in Uganda. Habitat and elevation are field records from our surveys. (C – Cultivation; R – Roadside; S – Seasonal wetland; T – Thicket; DWG – Degraded wetland grounds; R/B- Riverbank; O – Open grassland and F – Forest).

No.	SPECIES	HABITAT								Elevation (m)
		C	R	S	T	DWG	R/B	O	F	
1	*S. aculeatissimum* Jacq.		x							1350–2750
2.	*S. aethiopicum*	x								1248–1272
3	*S. aculeastrum* Dunal	x	x							1200–2500
4	*S. anguivi* Lam.	x	x							(40) 1000 -2200 (3100)
5	*S. campylacanthum* Hochst. ex A. Rich	x	x	x	x		x	x	x	0 - 2000 (2300)
6	*S. cerasiferum* Dunal		x							3000
7	*S. coagulans* Forssk		x					x		200–1500
8	*S. chrysotricum*		x							1607
9	*S. cyaneopupureum* De wild		x							800–1500
10	*S. dasyphyllum* Thonn	x	x							600–1600
11	*S. forskalii* Dunal (*S. albicaule*)			x						150–1300
12	*S. giganteum* Jacq							x		800–2450
13	*S. hastifolium* Hochst. Ex Dunal		x			x				200–1700
14	*S. lanzae* Lebrun and Stork		x							1200–2100
15	*S. macrocarpon* L.							x		1050–1700
16	*S. melongena*	x								1268
17	*S. tettense* K lotsch (*S. renschii*)		x		x					800–1400
18	*S. wrightii* Benth		x							800–1700

**Fig. 3:**
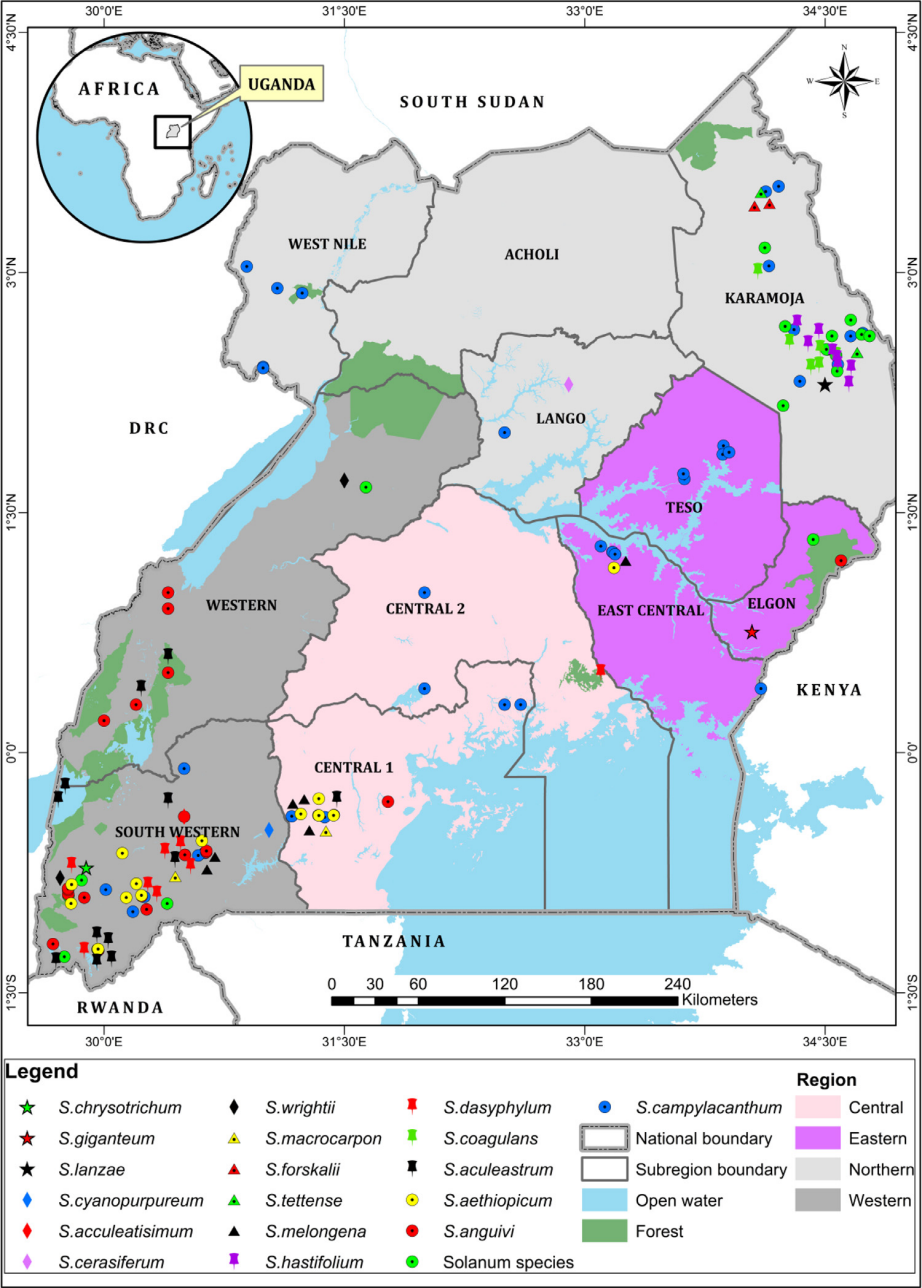
Species distribution map for 18 species of the Subgenus *Leptostemonum* in Uganda.

Occurrence points obtained as geo-coordinates from our field surveys were used to generate a species distribution map (Fig. 3) using ArcMap 10.7.1 software. The mapping units used for the distribution map were based on the regions of Uganda and sub-regions as defined in Table 3.

**Table 3:** Subregions of Uganda used for species distribution mapping.

Sub regions	Regions of Uganda			
	Northern (U1)	Western (U2)	Eastern (U3)	Central (U4)
	West Nile	Western	Teso	Central 1
	Acholi	South western	Elgon	Central 2
	Lango		East central	
	Karamoja			

## 4 Experimental Design, Materials and Methods

### 4.1 Materials and methods

#### 4.1.1 Study area

Field surveys were done in selected districts of Uganda. The sites were purposively selected based on distribution data that was gathered from Makerere University herbarium (MHU) and the Flora of Tropical East Africa, Solanaceae [5].

### 4.2 Field surveys

Sampling was done between July and December 2019 to collect presence data for the species of subgenus *Leptostemonum*. At each sampling site, herbarium voucher specimens were collected and the collection points geocoded using a hand-held Global Positioning System (GPS). Other field data including; habitat type, elevation, and name of locality were also recorded. Field identification of the specimens was guided by the use of the FTEA [5] and confirmation was at Makerere University herbarium. In total, 172 specimens were collected using standard methods as described in [6] then pressed, dried and deposited at MHU for name confirmation.

### 4.3 Species occurrence data and distribution mapping

Field survey locations’ data (geocoded with a ≤ 5M Germin S90 GPS) formed the main data set to generate a species distribution map using ArcMap 10.7.1 software. The mapping units used for the distribution map were based on the regions and sub-regions of Uganda.

## Limitations

Species growing in flooded areas (if present) were not captured due to failure to access such habitats because data was collected during heavy rains and also, insufficient sampling time.

## Ethics Statement

The authors have read and followed the ethical requirements for publication in Data in Brief and confirming that the current work does not involve human subjects, animal experiments, or any data collected from social media platforms.

## CRediT Author Statement

Carol Kawuma: Data collection, writing original draft preparation. Derick Serujongi: Data collection and analysis. Godwin Anywar: writing, reviewing and editing. Mary Namaganda: Supervision and data curation, Esther Katuura: Methodology*.*

## Data Availability

Diversity and distribution dataset for species of the Leptostemonum clade, genus Solanum in Uganda (Version 2) (Original data) (Mendeley Data)
